# Anxiety in Polish adult patients with inborn errors of immunity: a cross-sectional study

**DOI:** 10.3389/fpsyt.2024.1293935

**Published:** 2024-03-07

**Authors:** Kinga Grochowalska, Marcin Ziętkiewicz, Katarzyna Nowicka-Sauer, Mariusz Topolski, Ewa Więsik-Szewczyk, Aleksandra Matyja-Bednarczyk, Katarzyna Napiórkowska-Baran, Zbigniew Zdrojewski

**Affiliations:** ^1^ Department of Rheumatology, Clinical Immunology, Geriatrics and Internal Medicine, Faculty of Medicine, Medical University of Gdańsk, Gdańsk, Poland; ^2^ Department of Family Medicine, Faculty of Medicine, Medical University of Gdańsk, Gdańsk, Poland; ^3^ Department of Systems and Computer Networks, Faculty of Information and Communication Technology, Wrocław University of Science and Technology, Wrocław, Poland; ^4^ Department of Internal Medicine, Pneumonology, Allergology and Clinical Immunology, Central Clinical Hospital of the Ministry of National Defense, Military Institute of Medicine, Warsaw, Poland; ^5^ Outpatient Clinic for the Immunological and Hypercoagulable Diseases, Medical University of Kraków, Kraków, Poland; ^6^ Department of Allergology, Clinical Immunology and Internal Diseases, Ludwik Rydygier Collegium Medicum in Bydgoszcz, Nicolaus Copernicus University in Toruń, Bydgoszcz, Poland

**Keywords:** inborn errors of immunity, anxiety, illness perception, depression, sleep quality

## Abstract

**Background:**

Patients with inborn errors of immunity (IEI) experience recurrent infections, autoimmunity, and malignancies. Owing to repeated medical procedures, the need for constant treatment and surveillance, and the unpredictable course of the disease, patients with IEI are prone to develop mental health disorders, including anxiety. In this study, we aimed to assess the prevalence and level of anxiety symptoms in adult Polish patients with IEI and explore the determinants of anxiety in this group of patients.

**Methods:**

Data from 105 Polish patients with IEI were collected via the hospital anxiety and depression scale (HADS), brief illness perception questionnaire (B-IPQ), illness cognition questionnaire (ICQ), Pittsburgh sleep quality index (PSQI), and a questionnaire on general health and demographic data. For statistical analyses of data, the normality of distribution of quantitative data was assessed, and internal consistency of tests was investigated using Cronbach’s alpha coefficient; moreover, we performed the analysis of correlations and between-group differences, and path analysis to explore causal relationships. Significance was considered at *p* < 0.050.

**Results:**

Thirty-eight (36.2%) patients had anxiety symptoms (HADS-A ≥ 8); 14 (13.3%) patients had severe anxiety (score ≥ 11), and 24 (22.9%) had moderate anxiety (score of 8–10). Patients with poor sleep quality, higher pain frequency, younger age, and no fixed income had higher anxiety scores than others. Emotional and cognitive representations of illness were positively correlated with anxiety levels. Intense anxiety was related to more negative illness perception, higher helplessness, lower illness acceptance, and lower perceived benefits.

**Discussion:**

Anxiety is common in patients with IEI. However, results indicate that it is not related to a more severe course of IEI or several comorbidities, whereas, pain frequency and poor sleep quality were identified to be important clinical factors for anxiety. Because anxiety was related to negative illness perception, psychological therapy may apply to this group of patients.

## 1 Introduction

Inborn errors of immunity (IEI) comprise a heterogeneous group of inherited disorders affecting the immune system (1). Previously, IEI were known as primary immunodeficiency disorders. The clinical manifestations of IEI include increased susceptibility to infections, autoimmunity, autoinflammatory diseases, allergies, and malignancies (2). Patients with IEI experience a deterioration in their physical condition owing to chronic patterns and recurrent infections. Furthermore, owing to repeated medical procedures, the need for constant treatment and surveillance, the threat of malignancy, and the unpredictable course of the disease; patients are prone to developing mental health disorders, including anxiety and depression (3).

Patient-reported outcomes (PROs) are a type of outcomes that are used to assess the subjective health status from the patients’ perspective without physicians’ interpretation (4). PROs constitute an essential component of psychological mental health assessment because they capture a patient’s subjective experience of the disease or intervention, which may not be reflected in clinical endpoints (5). The implementation of PROs is faced with many obstacles in daily clinical practice (6). Nevertheless, they should be integrated into the clinical routine.

Anxiety is a mental state characterized by excessive fear and concern about potential situations (7). It may be caused by external or internal factors (8). Although anxiety is common in chronic diseases (9), it is not routinely assessed and is rarely treated (10). Recent scientific reports have revealed an increased level of anxiety in patients with IEI (11). However, to the best of our knowledge, the determinants of anxiety have not yet been investigated.

Illness perception defines patients’ beliefs about their disease. According to the Common Sense Model theory, each patient experiencing a disease creates his or her own representation of a condition (12). It comprises of cognitive and emotional representations (13). Cognitive representation comprises five dimensions: identity (patient’s description of illness), consequences (how an illness impacts patient’s life), cause (patient’s beliefs regarding the cause of a disease), timeline (length of the disease in patient’s belief), and cure or control (patient’s belief about the effectiveness of treatment and perceived control of the disease) (14). Emotional representation comprises concerns and negative emotions related to a disease. In addition, this model involves the patient’s understanding of a disease (15).

Illness perception influences emotional reactions in patients and how they cope with daily situations (16). In recent years, interest in the assessment of illness perception has increased. As a potentially modifiable aspect of chronic diseases, illness perception may improve clinical outcomes, or vice versa, leading to even higher mortality (17, 18).

Patients with chronic diseases may experience a range of negative emotions, including anxiety, depression, irritation, and anger, which can interfere (19). In this study, we aimed to investigate these problems in our patients. Previously, patients with IEI were mainly assessed for the prevalence of depression. However, some studies revealed that anxiety is potentially more prevalent than depression among patients with IEI (20), and it is often overlooked in clinical practice. In our previous study (3), which included a part of the current study (n=92), anxiety symptoms were frequent. Therefore, we expanded our sample (n=105) and considered anxiety as a dependent variable to perform a more precise analysis of factors, including anxiety levels, the association of anxiety levels with sleeping disorders, and pain. Moreover, we have evaluated the frequency of pain to determine whether chronic pain is associated with increased anxiety levels.

Anxiety disorders and high levels of anxiety are related to sleeping problems, leading to altered sleep quality. Sleep disorders are common among patients with chronic medical conditions (21). Many longitudinal studies revealed bidirectional correlations between sleep quality and anxiety (22). Previously (3), using a multiple linear regression model, we identified anxiety symptoms as an important predictor of poor sleep quality. Therefore, we decided to further analyze the association between anxiety and sleep quality in the current manuscript.

In this study, we assessed the prevalence of anxiety symptoms and anxiety levels in adult Polish patients with IEI. We also explored the clinical, sociodemographic, and psychological determinants of anxiety in this patient group. To the best of our knowledge, this is the first study to assess illness perception in patients with IEI.

## 2 Materials and methods

### 2.1 Study design and sample

Adult patients (≥ 18 years) diagnosed with IEI according to the diagnostic criteria of the European Society for Immunodeficiencies (23) were recruited from four Polish clinical centers in Bydgoszcz, Gdańsk, Kraków, and Warszawa between February 2021 and December 2022. In total, 120 eligible individuals were selected for this study. Eight patients refused to participate in the study. Seven individuals were excluded because they did not complete the questionnaires. Finally, 105 participants, including 55 women (52.4%) and 50 men (47.6%) with a mean age of 42.16 ± 14.1 years were recruited in this study.

In this cross-sectional observational study, data were collected via the hospital anxiety and depression scale (HADS), brief-illness perception questionnaire (B-IPQ), illness cognition questionnaire (ICQ), and Pittsburgh sleep quality index (PSQI).

Additionally, the survey included demographic questions regarding age, sex, professional activity, and residential status, and clinical data, including comorbidities and type of treatment, with special emphasis on immunoglobulin replacement therapy.

To describe pain, we assessed its frequency with the following questions: How often did you experience pain during the last 3 months? Possible answers were: “not at all”, “for a few days”, “more than 30 days”, or “almost every day”.

To determine the disease activity, we evaluated the following variables: the number of infections in the last 3 months, antibiotic administration in the last 3 months, and hospitalizations (excluding those for immunoglobulin administration) in the last 3 months.

All patients provided written informed consent before the study. The study design was approved by the Independent Bioethics Commission for Research of the Medical University of Gdańsk (Number: 422/2017).

### 2.2 Hospital anxiety and depression scale

The HADS consists of 14 questions, including seven for the anxiety subscale (HADS-A), and seven for the depression subscale (HADS-D), which are rated on a 0–3 response Likert scale. The maximum score for each subscale is 21 points; scores < 8 indicate a normal result, whereas, 8–10 points indicate moderate anxiety or depressive symptoms, and ≥ 11 presents severe depressive or anxiety symptoms (24). In the present study, we used a modified version of HADS since we sought to assess a wider range of emotions and modified HADS allows to assess irritability. HADS-M contains the following items: (I) “It happened that during the last week, I exploded with anger”; (II) “It happened that I got upset internally”. Patients assess their irritability level using a Likert scale with scores ranging from 0 (“not at all”) to 6 points (“frequently”), where a higher score indicated a higher level of irritability. The modified HADS has satisfactory psychometric properties (19). In this study, Cronbach’s alpha coefficients for depression, anxiety, and irritability subscales of HADS were 0.86, 0.82, and 0.84, respectively.

### 2.3 Brief illness perception questionnaire

The B-IPQ is widely used to measure cognitive and emotional representations of illnesses in patients with various chronic diseases. It consists of eight items rated on a 0-to-10 response scale. Additionally, it includes an open-ended question regarding patient-perceived causes of the disease (13, 14). Each of the eight items refers to one of the following illness perception dimensions: consequences, timeline, personal control, treatment control, identity, coherence, concern, and emotional response. Moreover, emotional and cognitive representations scores can be calculated. A higher total B-IPQ score indicates a more negative illness perception (13, 14). A previous study, conducted among patients with chronic somatic diseases, validated the psychometric properties of the Polish version of the B-IPQ (13). In the present study, Cronbach’s alpha was 0.75.

### 2.4 Illness cognition questionnaire

The ICQ is an 18-item instrument that measures three dimensions of illness cognition: helplessness, acceptance, and perceived benefits (25, 26). Helplessness indicates the degree of powerlessness and hopelessness experienced by patients regarding their condition and its consequences in their lives. Acceptance refers to the degree of adaptation and integration of a condition into reality. Perceived benefits indicate the degree of positive outcomes or opportunities that patients perceive in their conditions. Each item is rated on a 4-point Likert scale ranging from 1 (not at all) to 4 (completely). The points for each subscale are added and range from 6 to 24 (25, 27). Higher subscale scores indicate higher, helplessness, acceptance, and perceived benefits. In the original study (25), Cronbach’s alpha ranged from 0.84 to 0.90. The current study involving patients with IEI exhibited satisfactory internal consistency of all three subscales (Cronbach’s alpha coefficients: 0.88 for helplessness, 0.87 for acceptance, and 0.83 for perceived benefits).

### 2.5 Pittsburgh sleep quality index

The PSQI is a self-rated questionnaire that measures sleep quality and disturbances over one month (28). It comprises 19 items grouped into seven domains: subjective sleep quality, sleep latency, sleep duration, habitual sleep efficiency, sleep disturbances, use of sleeping medication, and daytime dysfunction. Each domain is scored from 0 to 3, with higher scores indicating poorer sleep quality or more sleep-associated problems. The sum of the seven domain scores yields the global PSQI score, which ranges from 0 to 21. A global PSQI score ≥5 indicates poor sleep quality (29). The internal consistency of the PSQI assessed using Cronbach’s alpha coefficient was 0.83 (28). The Polish version of the scale was used in a study that involved Polish patients with systemic lupus erythematosus (SLE) (30). In the present study, Cronbach’s alpha was 0.82.

### 2.6 Statistical analysis

Descriptive statistics (mean, standard deviation, median, maximal, and minimal value for quantitative variables, and number and percentage for categorical variables) were analysed to summarize the study outcomes. The internal consistency of the scales was verified using the Cronbach’s alpha coefficient. The normality of the distribution of the variables was verified using the Shapiro–Wilk test. Continuous variables were analyzed using Student’s *t*-, Mann–Whitney *U*, and Kruskal–Wallis tests with *post-hoc* Dunn analysis, in case of multiple comparisons. Associations between anxiety and categorical variables were assessed via between-group comparisons. Categorical variables were analyzed using the chi-square or Fisher exact tests. The associations between anxiety level and quantitative independent variables were assessed using Spearman’s rho correlation coefficient. Path analysis was used to examine both direct effects, which are the direct influences of one variable on another, and indirect effects, which result from the path between two variables through one or more other variables. In the diagram, the variables are represented as nodes and the directional relationships between them are represented with arrows. The path analysis was conducted using the AMOS package. Significance was considered at *p* < 0.050. Statistical analysis was performed using the STATISTICA software (version 13; TIBCO Software Inc., Palo Alto, CA, USA).

## 3 Results

In the statistical analysis, descriptive statistics were first calculated for categorical variables (**Table 1**) and quantitative variables (**Table 2**).

**Table 1:** Frequency and percentage statistics for categorical variables in the studied sample of patients with inborn errors of immunity (n=105).

Variable	*n* (%)
Sex	
Women	55 (52.4)
Men	50 (47.6)
Education	
Primary	7 (6.7)
Vocational	8 (7.6)
Higher	40 (38.1)
Secondary	50 (47.6)
Work status	
Active worker	59 (56.2)
Non-active worker	36 (34.3)
Student	10 (9.5)
Income	
No income	12 (11.4)
Fixed income	93 (88.6)
Residential status	
City ≥ 100 000 inhabitants	44 (41.9)
City 50 000–100 000 inhabitants	15 (14.3)
City ≤ 50 000 inhabitants	19 (18.1)
Village	27 (25.7)
Pain frequency in the previous 3 months	
Not at all	19 (18.1)
For few days	46 (43.8)
More than 30 days	8 (7.6)
Almost everyday	32 (30.5)
IEI treatment	
Without treatment	5 (4.8)
SCIg	82 (78.1)
IVIg	15 (14.3)
Other than Ig replacement therapy	3 (2.9)
Hospitalization in the previous 3 months (for reasons other than immunoglobulin administration)	
Yes	19 (18.1)
No	86 (81.9)
Administration of antibiotics in the previous 3 months	
Yes	72 (68.6)
No	33 (31.4)
Anxiety (HADS)	
No anxiety symptoms (< 8 points)	67 (63.8)
Moderate anxiety symptoms (8–10 points)	24 (22.9)
Severe anxiety symptoms (≥ 11 points)	14 (13.3)
Sleep quality (PSQI)	
Good (< 5 points)	57 (54.3)
Poor (≥ 5 points)	48 (45.7)

IEI, inborn errors of immunity; Ig, immunoglobulin; IVIg, intravenous immunoglobulin; SCIg, subcutaneous immunoglobulin; HADS, hospital anxiety and depression scale; PSQI, Pittsburgh sleep quality index; n, number.

**Table 2:** Descriptive statistics for quantitative variables in the studied sample of patients with inborn errors of immunity (n=105).

Variable	Mean (SD)	Median (min–max)
**Age (years)**	42.16 (14.1)	42.00 (18–76)
**Duration of IEI (years)**	11.78 (10.4)	9.00 (0–57)
**Number of infections in the previous 3 months**	1.31 (3.2)	0.00 (0–29)
**Number of chronic diseases**	2.13 (2.6)	1.00 (0–15)
HADS		
Anxiety	5.83 (4.3)	5.00 (0–16)
Depression	4.39 (4.0)	3.71 (0–14)
Irritability	2.96 (1.7)	3.00 (0–6)
B-IPQ		
Consequences	5.96 (2.7)	6.00 (0–10)
Timeline	9.26 (2.0)	10.00 (1–10)
Personal control	6.82 (2.4)	7.00 (0–10)
Treatment control	8.56 (1.9)	9.00 (2–10)
Identity	5.96 (2.8)	7.00 (0–10)
Concern	5.57 (2.9)	6.00 (0–10)
Understanding	7.95 (2.1)	8.00 (2–10)
Emotional response	4.92 (3.0)	5.00 (0–10)
Cognitive representation	27.85 (7.5)	28.00 (10–49)
Emotional representation	10.50 (5.4)	11.00 (0–20)
Illness perception – Total	38.34 (12.1)	39.00 (10–67)
ICQ		
Helplessness	11.97 (4.1)	12.00 (6–24)
Acceptance	17.67 (3.8)	18.00 (11–24)
Perceived benefits	16.12 (4.2)	16.00 (7–24)

IEI, inborn errors of immunity; HADS, hospital anxiety and depression scale; B-IPQ, the brief-illness perception questionnaire; ICQ, illness cognition questionnaire; SD, standard deviation; min, minimum; max, maximum.

Anxiety symptoms (HADS-A ≥ 8) were detected in 38 (36.2%) patients; 14 (13.3%) patients reported a score ≥ 11 indicating severe anxiety symptoms, and 24 (22.9%) patients had moderate anxiety symptoms (**Table 1**).

PSQI-based analysis revealed that 57 patients (54.3%) had good sleep quality, whereas, 48 patients (45.7%) reported poor sleep quality (**Table 1**). The majority of patients (n=86, 81.9%) experienced general pain. Only 18.1% of patients (n = 19) did not report pain in the previous 3 months, whereas 30.5% of the patients (n = 32) reported pain almost every day (**Table 1**).

The mean age was 42.16±14.1 years, the mean IEI duration was 11.78 ± 10.4 years; the mean number of chronic disease was recorded to be 2.13 ± 2.6. (**Table 2**).

The correlation analysis was performed to explore the association between anxiety levels and quantitative variables (**Table 3**). Age and number of chronic diseases were not significantly correlated with anxiety levels (**Table 3**). Moreover, disease-related factors, such as IEI duration and the number of infections in the previous 3 months, were not correlated with anxiety levels (**Table 3**).

**Table 3:** Matrix of Spearman’s rho correlation coefficients between the anxiety level (dependent variable) and independent variables in the studied sample of patients with inborn errors of immunity (n=105).

Variable	Anxiety levels	
	Spearman’s rho	*p*
Age (years)	−0.060	0.542
Duration of IEI (years)	−0.143	0.146
Number of infections in the previous 3 months	0.026	0.796
Number of chronic diseases	0.153	0.119
HADS		
Depression	0.721*	< 0.001
Irritability	0.497*	< 0.001
B-IPQ		
Consequences	0.273*	0.005
Timeline	−0.036	0.713
Personal control	−0.387*	<0.001
Treatment control	−0.095	0.337
Identity	0.246*	0.011
Concern	0.389*	<0.001
Understanding	−0.265*	<0.001
Emotional response	0.405*	<0.001
Cognitive representation	0.372*	<0.001
Emotional representation	0.425*	<0.001
Illness perception – Total	0.416*	<0.001
ICQ		
Helplessness	0.208*	0.033
Acceptance	−0.379*	<0.001
Perceived benefits	−0.284*	0.003

IEI, inborn errors of immunity; HADS, hospital anxiety and depression scale; BIPQ, the brief-illness perception questionnaire; ICQ, illness cognition questionnaire; *p < 0.050.

B-IPQ revealed that, among the illness perception dimensions, statistically significant positive correlations were detected between anxiety and consequences (*r* = 0.273; *p* = 0.005), identity (*r* = 0.246; *p* = 0.011), concern (*r* = 0.389; *p* < 0.001), and emotional response (*r* = 0.405; *p* < 0.001). Significant negative correlations were found between anxiety and personal control (*r* = −0.387; *p* < 0.001), as well as understanding (*r* = −0.265; *p* < 0.001); whereas emotional representation (*r* = 0.425; *p* < 0.001), cognitive representation (*r* = 0.372; *p* < 0.001), and total illness perception score (*r* = 0.416; *p* < 0.001) showed significant positive correlation with anxiety (**Table 3**).

In the ICQ results, we noted a positive correlation between anxiety and helplessness (*r* = 0.208; *p* = 0.033), and a negative correlation between anxiety and acceptance (*r* = −0.379; *p* < 0.001) and perceived benefits (*r* = −0.284; *p* = 0.003) (**Table 3**).

Significant positive correlations of anxiety with depression (*r* = 0.721; *p* < 0.001), irritability (*r* = 0.497; *p* < 0.001) were detected (**Table 3**).

The analysis of relationship between anxiety and categorical variables was performed using group comparisons (**Table 4**).

**Table 4:** Differences in anxiety levels in relation to independent categorical variables in the studied sample of patients with inborn errors of immunity (n=105).

Variable	Anxiety levels		Test
	Mean (SD)	Median (min–max)	
Sex			
Women	6.15 (4.5)	6.00 (0–16)	Mann–Whitney U test
Men	5.48 (4.1)	5.00 (0–16)	
Education			
Primary	7.57 (6.4)	6.00 (1–16)	*H* = 0.943; *p* = 0.624Kruskal–Wallis test, *post-hoc* Dunn
Vocational	4.75 (4.7)	4.00 (0–14)	
Higher	5.58 (4.3)	4.00 (0–16)	
Secondary	5.96 (4.0)	6.00 (0–16)	
Work status			
Active worker	5.49 (4.11)	5.5 (0–16)	*H* = 0.716; *p* = 0.699Kruskal–Wallis test, *post-hoc* Dunn
Non-active worker	6.17 (4.45)	5.0 (0–15)	
Student	6.60 (5.30)	5.5 (0–16)	
Income			
No income	8.92 (5.04)	8.5 (1–16)	*Z* = −2.240; *p* = 0.025Mann–Whitney *U* test
Fixed income	5.43 (4.08)	5.0 (0–16)	
Residential status			
City ≥ 100 000 inhabitants	6.18 (4.34)	6.50 (0–16)	*H* = 5.129; *p* = 0.163Kruskal–Wallis test, *post-hoc* Dunn
City 50 000–100 000 inhabitants	5.40 (4.79)	4.00 (0–16)	
City ≤ 50 000 inhabitants	7.21 (4.59)	7.00 (0–16)	
Village	4.52 (3.62)	4.00 (0–15)	
Pain frequency in the previous 3 months			
Not at all (0)	2.89 (2.83)	2.00 (0–9)	*H* = 16.324; *p* < 0.001Kruskal–Wallis test, *post-hoc* Dunn*[3] > [0]; [3] > [1]; [2] > [0]
For a few days (1)	5.57 (3.88)	5.00 (0–16)	
More than 30 days (2)	6.13 (5.52)	7.00 (0–15)	
Almost every day (3)	7.88 (4.43)	8.00 (0–16)	
IEI treatment			
Without treatment	5.60 (5.595)	4.00 (1–15)	*H* = 4.004; *p* = 0.261Kruskal–Wallis test, *post-hoc* Dunn
SCIg	5.83 (4.48)	5.00 (0–16)	
IVIg	6.67 (2.85)	8.00 (2–10)	
Other than Ig replacement therapy	2.00 (3.46)	0.00 (0–6)	
Hospitalization in the previous 3 months (for reasons other than immunoglobulin administration)			
Yes	6.05 (4.42)	6.00 (0–15)	*Z* = −0.314; *p* = 0.754Mann–Whitney *U* test
No	5.78 (4.33)	5.00 (0–16)	
Administration of antibiotics in the previous 3 months			
Yes	5.89 (4.13)	5.00 (0–16)	*Z* = −0.468; *p* = 0.640Mann–Whitney *U* test
No	5.70 (4.79)	5.00 (0–16)	
Sleep quality (PSQI)			
Good (< 5 points)	3.91 (3.51)	4.00 (0–16)	*Z* = −5.031; *p* < 0.001Mann–Whitney *U* test
Poor (≥ 5 points)	8.10 (4.12)	8.50 (0–16)	

IEI, inborn errors of immunity; Ig, immunoglobulin; IVIg, intravenous immunoglobin; SCIg, subcutaneous immunoglobulin; PSQI, Pittsburgh sleep quality index; SD, standard deviation; min, minimum; max, maximum.

* (3) > (0), patients, reporting pain experienced almost every day in the previous 3 months, had significantly higher levels of anxiety than patients declaring no pain; (3) > (1), patients declaring pain experienced almost every day in the previous 3 months had significantly higher level of anxiety than patients experiencing pain for a few days during the previous 3 months; (2) > (0), patients declaring pain occurring more than 30 days in the previous 3 months had significantly higher level of anxiety than patients declaring no pain.

Among the sociodemographic factors, only income exhibited a correlation with anxiety; patients with no income had significantly higher anxiety levels compared to those with fixed incomes (scores of 8.92 ± 5.04 vs. 5.43 ± 4.08; *p* = 0.025). Sex, education, professional activity, and domicile were not associated with anxiety (**Table 4**).

Among clinical features, only pain frequency was related to anxiety levels. Patients with a higher pain frequency had a higher anxiety score (*p* < 0.001). Hospitalization in the previous 3 months, antibiotic administration, and IEI treatment were not related to anxiety (**Table 4**).

Patients with poor sleep quality had a higher score for anxiety than those with good sleep quality (8.10 ± 4.12 vs. 3.91 ± 3.51; *p* < 0.001) (**Table 4**). The majority of patients (n = 41, 82.0%) with good sleep quality did not experience anxiety.

**Figure 1:**
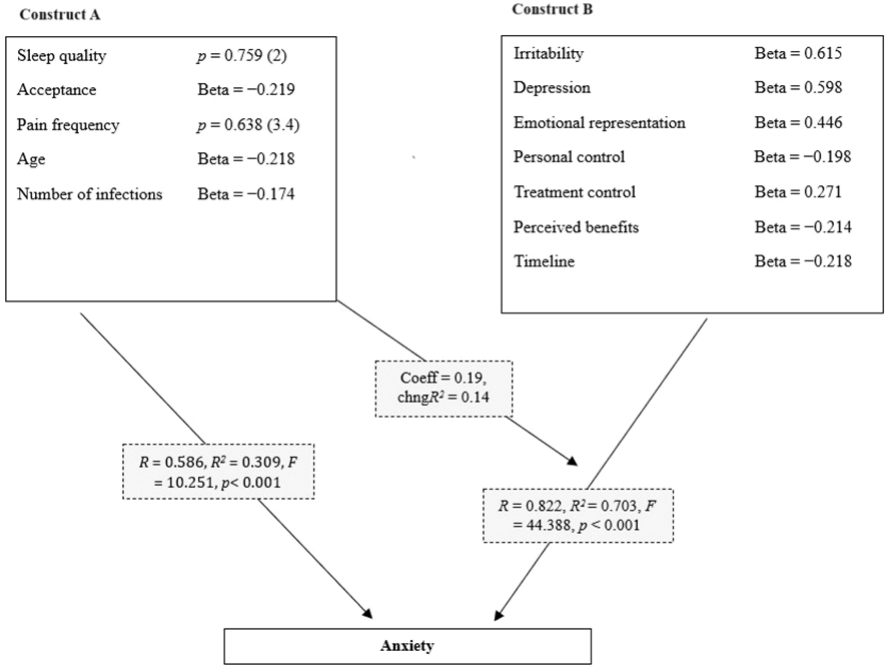
Path analysis of potential causal relationships between anxiety level and independent variables in the studied sample of patients with inborn errors of immunity (n=105). (RMSEA = 0.038; CFI = 0.951; CMIN = 44.287; *p* < 0.01). The following statistics were used in the moderation analysis. ;*R*: Correlation before moderation; *R^2^*: Coefficient of determination before moderation; Change in *R^2^*: Change in the coefficient of determination due to moderation; Coeff - coefficient of moderation analysis; p: significance of the moderator’s influence on the model; RMSEA: Model fit index.

To explore the causal relationship between studied variables, path analysis was performed. The obtained value of the root mean square error of approximation (RMSEA) parameter (0.038; *p* < 0.050), indicated a well-fitting model and was statistically significant. Higher scores for irritability (Beta = 0.615), depression (Beta = 0.598), and emotional representation (Beta = 0.446), as well as lower scores for personal control (Beta = −0.198), higher treatment control (Beta = 0.271), lower perceived benefits (Beta = −0.214), and lower timeline (Beta = −0.218), were associated with higher anxiety scores. This model explains 70.3% of the variance. These results were further reinforced by moderator A, which consists of the following relationships: higher likelihood of poor sleep quality (*p* = 0.759) and lower acceptance (Beta = −0.219), higher frequency of pain (“at least 1 month in previous 3 months” and “almost every day in previous 3 months”; *p* = 0.638), younger age (Beta = −0.218), and lower number of infections (Beta = −0.174). These relationships enhance the association between construct B and the dependent variable by approximately 14%. On the contrary, construct A alone explained 30.9% of the total variance in anxiety (**Figure 1**).

## 4 Discussion

### 4.1 Patients’ emotional state

Patients with chronic diseases might be affected by psycho-emotional disorders. They may experience psychological distress, such as fear, anger, helplessness, powerlessness, and a range of negative affective states. In current attempts to provide the highest compliance, there has been a constant improvement in medical therapies and treatment protocols. Hence, assessing patient-related outcomes, including emotional state and illness perception could be indispensable.

The subjective image of a disease is reported to be more relevant to the patient than the measured disease severity (31). While exploring other factors related to the well-being of patients with IEI, we decided to evaluate the frequency and level of anxiety and the factors associated with enhanced levels of anxiety.

### 4.2 Anxiety

Among the patients included in this study, 36.2% had anxiety symptoms according to HADS, with 13.3% and 22.9% having severe and moderate levels of anxiety, respectively. A review of the literature reveals a strong association between anxiety and chronic diseases (32). However, anxiety is frequently overlooked and inadequately managed; therefore, appropriate diagnosis and intervention are required. Owing to a lack of awareness and underestimation of the prevalence of anxiety, many patients do not receive proper treatment. Anyfanti et al. studied 514 patients with rheumatic disease and noted anxiety and depression in 30.8% and 21.8% of the patients, respectively. However, anti-anxiety medications were prescribed to only 12.1% of the patients with anxiety symptoms (10).

Improper treatment of anxiety and depressive symptoms may lead to the failure of somatic disease treatment. Matcham et al. conducted a longitudinal study to investigate the association between symptoms of anxiety and depression, and the response to prednisolone. Interestingly, patients with symptoms of anxiety or depression at the baseline also showed a 50% reduction in the effects of prednisolone treatment compared to patients with no symptoms of anxiety or depression at the baseline (33). Owing to the worsening mental health and diminishing treatment efforts, anxiety and depression are associated with increased mortality in patients with rheumatoid arthritis (34), chronic obstructive pulmonary disease, and heart failure (35). Hence, studies exploring the determinants of negative emotionality, especially the modifiable ones, are crucial, since they allow for the implementation of targeted and effective interventions.

Only a few studies on anxiety in patients with IEI have been reported. In a pilot study, Heath et al. detected that anxiety was more frequent in patients with IEI (n = 33) than in the general population. Risk factors for significantly elevated anxiety include poor health and a lack of refreshing sleep (11).

Sower et al. identified significantly higher anxiety scores in 292 patients with IEI compared to the normative values. Besides enhanced anxiety and depression, patients with IEI exhibited a greater degree of perceived memory impairment or brain fog (36). Previously, we revealed that 38% of patients with IEI had anxiety symptoms, which were more frequent than depression symptoms (3).

In this study, no correlations were detected between the activity of the disease and the anxiety levels. Pain was considered the only important clinical feature. Thus, clinical data alone are insufficient for determining the anxiety levels. Among sociodemographic variables, only age and income status were related to anxiety levels.

In our group, patients who had fewer infections and did not receive antibiotics in the previous 3 months, a sign of a more benign course of IEI, had higher levels of anxiety. Anxiety level was not related to disease duration, number of comorbidities, or number of hospitalizations. The only significant clinical variable was pain frequency. Patients with higher pain frequency had higher levels of anxiety. Smith et al. conducted an 11-week study to examine the influence of anxiety and depression on pain in women with arthritis. They revealed that both anxiety and depression predicted higher levels of pain in the same and the following weeks; however, the impact of anxiety was stronger than that of depression (37). Contrarily, Kosson et al. examined 1025 patients treated in pain clinics and revealed that the level of anxiety can be determined by pain intensity. A higher pain intensity was associated with higher anxiety levels (38). Although the causal relationship between pain and anxiety remains unclear, it appears to be bidirectional. Further longitudinal studies are required to determine the direction of this association. According to the findings described above, it is crucial to assess the anxiety status of patients experiencing pain.

Our study revealed that patients with poor sleep quality had higher anxiety scores than those with good sleep quality. Our results are consistent with those reported by Heath et al. who revealed that a lack of refreshing sleep was a risk factor for significantly elevated anxiety in patients with IEI (11).

### 4.3 Illness perception and anxiety

The current study revealed significant associations between anxiety and several psychological variables in both the correlation and path analyses. More intense anxiety was associated with higher levels of depression and irritability, indicating a more complex multidimensional picture of negative emotionality. Moreover, more negative emotional and cognitive representations of illness, including perceived severe consequences and symptoms, perceived personal uncontrollability of the disease and lower understanding as well as higher helplessness, lower acceptance of the disease and lower perceived benefits related to the disease, were significantly associated with heightened levels of anxiety. Despite the high perceived treatment effectiveness and low frequency of infections, patients had a very high average score (9.2) in the timeline dimension, with a median score of 10 points. These results indicate patients’ awareness of the chronic course of their disease.

A perusal of the literature provides insights into the association between illness perception and anxiety in many chronic conditions (18). The results of the present study are comparable to previous reports that revealed that a more negative perception of illness is associated with higher anxiety (39). Similarly, an association of helplessness with anxiety and depression was reported in patients with systemic lupus erythematosus (40). In a study conducted among patients with type 1 diabetes, those who perceived disease benefits had more positive illness perception and lower levels of depression, anxiety, and irritability (41).

Furthermore, the impact of illness perception on emotional states, including depression, anxiety, and mental well-being, was reported in patients with heart failure (42, 43), fibromyalgia (44), and unruptured intracranial aneurysm (45).

### 4.4 Therapies targeting illness perception and anxiety

The results of the present study indicate that targeted interventions focusing on maladaptive illness perception are potentially important. Cognitive behavioral therapy resulted in a more positive illness perception and less anxiety in patients with unruptured intracranial aneurysms (45). The strong association between the cognitive model of illness and its emotional dimensions observed in our study justifies the suggestion that psychological therapies should address both cognitive illness representation and regulation of emotions (43, 44). Hence, interventions focused on illness perception should be considered as a part of a holistic medical approach that leads to improved health outcomes (18, 36).

### 4.5 Limitations

Despite careful planning, this study had some limitations. Anxiety symptoms were assessed using the HADS and were not verified according to the DSM-5 classification. Psychiatric examination is the best method to evaluate mental disorders. However, conducting a psychiatric examination for every patient would be difficult. Therefore, primary screening with the usage of standardized questionnaires seems justified in search for patients at higher risk of a mental disorder.

Furthermore, the patients were asked to provide their chronic disease information; however, we did not investigate mental disorders other than those mentioned by the patients. Patients provided data concerning general health and medication administration, which were not further verified. Nearly one-third of the patients did not name the administrated medicines; therefore, this variable was excluded from the analysis. Unlike a longitudinal study, this observational study did not support the observation of progression; however, the conducted path analysis led to preliminary conclusions about a causal relationship. Prospective, further interventional studies can shed more light on this topic.

### 4.6 Concluding remarks

Our study revealed that anxiety is common in patients with IEI. Anxiety was not related to a more severe course of IEI or the number of comorbidities, whereas pain and poor sleep quality were important clinical factors associated with high anxiety levels. Therefore, proper assessment and subsequent treatment of pain and sleep disorders may be beneficial.

We cannot infer the level of anxiety based on clinical data, as subjective illness perception has proven to be the most crucial factor. Our study revealed that anxiety is related to negative illness perception and, therefore, psychological therapy can be beneficial to patients with IEI. Future studies with a larger group of patients could further reveal the relationship between negative emotionality, illness representation, and clinical features of immunodeficiencies.

## Data availability statement

The raw data supporting the conclusions of this article will be made available by the authors, without undue reservation.

## Ethics statement

The studies involving humans were approved by Independent Bioethics Commission for Research of the Medical University of Gdańsk (Number: 422/2017). The studies were conducted in accordance with the local legislation and institutional requirements. The participants provided their written informed consent to participate in this study.

## Author contributions

KG: Conceptualization, Data curation, Formal analysis, Investigation, Methodology, Project administration, Resources, Writing – original draft, Writing – review & editing. MZ: Conceptualization, Data curation, Formal analysis, Investigation, Methodology, Project administration, Resources, Writing – original draft, Writing – review & editing. KNS: Conceptualization, Supervision, Writing – original draft. MT: Methodology, Writing – review & editing. EWS: Data curation, Writing – review & editing. AM: Data curation, Writing – review & editing. KN-B: Data curation, Writing – review & editing. ZZ: Supervision, Writing – review & editing.
